# Heterologous Production of Antimicrobial Peptides in Yeast Allows for Massive Assessment of the Activity of DNA-Encoded Antimicrobials In Situ

**DOI:** 10.32607/actanaturae.27355

**Published:** 2025

**Authors:** S. O. Pipiya, A. O. Ivanova, Yu. A. Mokrushina, I. E. Eliseev, A. G. Gabibov, I. V. Smirnov, S. S. Terekhov

**Affiliations:** Shemyakin–Ovchinnikov Institute of Bioorganic Chemistry of the Russian Academy of Sciences, Moscow, 117997 Russian Federation; Faculty of Chemistry, Lomonosov Moscow State University, Moscow, 119991 Russian Federation

**Keywords:** antimicrobial peptides (AMPs), yeast Pichia pastoris, heterologous production, template search, protegrin-1, thanatin

## Abstract

Antibiotic resistance threatens global healthcare. In clinical practice, conventional antibiotics are becoming gradually less effective. Moreover, the introduction of new antimicrobial agents into clinical practice leads to the emergence of resistant pathogenic strains within just a few years. Hence, the development of platforms for massive creation and screening of new antimicrobial agents is of particular importance. Massive parallel screening will greatly reduce the time required to identify the most promising drug candidates. Meanwhile, DNA-encoded antimicrobial agents offer unique opportunities for the high-throughput development of new antibiotics. Here, the yeast *Pichia pastoris *was engineered to produce a panel of antimicrobial peptides (AMPs), followed by high-throughput screening of AMP producers that inhibit bacterial growth *in situ*. Yeast clones producing thanatin and protegrin-1 exhibited the highest level of antimicrobial activity among the panel of AMPs under investigation. The production level of recombinant thanatin was significantly higher than that of protegrin-1, which correlates with its low toxicity. The designed technique of massive assessment of the activity of DNA-encoded antimicrobial agents enables the identification of drug candidates with an increased therapeutic index. Further development of methods for a rational design of artificial diversity in AMPs, followed by deep functional profiling of antimicrobial activity, will yield new AMPs with improved therapeutic characteristics.

## INTRODUCTION

The spread of antibiotic resistance renders conventional broad-spectrum antibiotics less effective, thus limiting treatment options for bacterial infections [1]. Furthermore, there is growing concern over the development of cross-resistance [2, 3]. Therefore, in order to tackle the rapid pace of microbial adaptation to drugs, it is necessary to expand the spectrum of potential antibiotics and increase their screening scale [4, 5].

Natural sources are a vast reservoir of compounds exhibiting antimicrobial activity; however, the search for and production of these compounds is often confined to culturable and highly abundant microbial strains [6]. In turn, this raises the problem of rediscovering already known antibiotics. This problem can be addressed by ultra-high throughput screening of samples from various sources [7], which allows one to identify compounds of different chemical nature. However, chemical synthesis or cultivation of the antibiotic producer and production of the target substance for subsequent experiments related to its modification is required to further fine tune the properties of these antibiotics.

Antimicrobial peptides (AMPs) are a promising class of alternative antibiotics [8]. Natural antimicrobial peptides active against Gram-negative bacteria are of exceptional interest in the context of combating the spread of hospital-acquired infections and antibiotic resistance [9]. The advantages of AMPs are that they are genetically encoded and have a simple biosynthetic pathway, which can be adapted for heterologous protein production. This makes it possible to easily make structural modifications and simplifies the procedure for screening and improving the pharmacological properties of AMPs. Analysis of peptide databases allows one to search across natural sources for new AMPs [10]. The metagenomic and proteomic data can also be analyzed for this purpose to identify potential AMPs and then test their antimicrobial activity [11, 12].

**Fig. 1:**
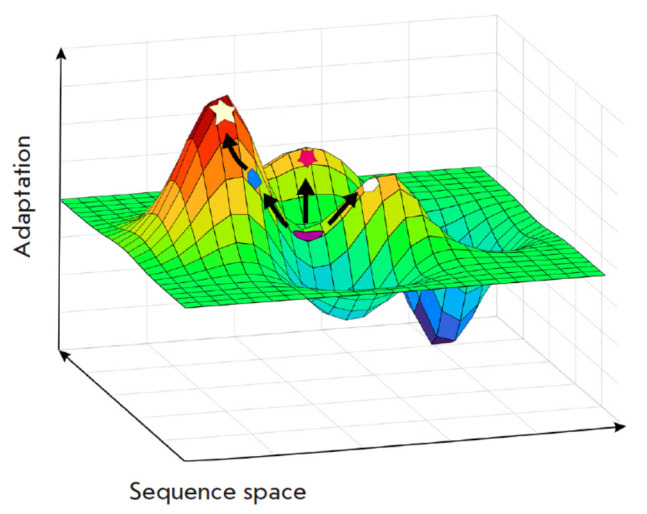
Schematic representation of directed evolution in the adaptation vs. sequence space coordinates

Another approach is to utilize artificial molecular diversity based on a rational design and *de novo* approaches. The libraries of AMP variants are constructed and tested using the chemical synthesis [13], phage display [14], or yeast display technologies [15].* De novo *design of AMPs using artificial intelligence and neural networks have recently witnessed extensive development [16]. Many of these approaches rely on directed evolution methods, which mimic the natural process in designing novel molecules. Choosing the starting point for generating a novel molecule is crucial in navigating the surface of the evolutionary landscape (*Fig. 1*). This will make it possible to analyze various directed evolutionary paths and identify the optimal variants. This setting of initial experimental conditions reduces the risk of following an evolutionary path that leads to a dead end.

Cytotoxicity and the mechanisms of action of AMPs are currently being assessed in artificial systems using vesicles, liposomes, and synthetic membranes as model membranes [17]. These methods allow one to roughly estimate how peptides interact with bacterial or eukaryotic membranes; however, they are conducted after the initial steps of antimicrobial activity screening and active peptide selection. AMP production in eukaryotic cells can be employed, and the antimicrobial activity of the producer strain can be assessed directly to reduce the number of screening steps and increase the throughput in research. Potentially cytotoxic peptides will not have a high yield in this heterologous system or will significantly affect the growth of the antibiotic-producer population. Hence, the number of variants can be minimized and allow one space to further investigate the properties of the selected AMPs more thoroughly. This approach can significantly accelerate the analysis.

The yeast *Pichia pastoris *is an interesting heterologous producer host in this regard, as it exhibits all the features of a eukaryotic cell, an affords the ability to introduce post-translational modifications, with a growth rate comparable to that of bacteria and a high level of recombinant protein production. [18].

The present study aims to investigate the features of heterologous AMP production in yeast and the application of recombinant technologies to template search for engineering novel antimicrobial peptides.

## EXPERIMENTAL

**Bacterial and yeast strains**

The methylotrophic yeast *P. pastoris *GS115 (Invitrogen, USA) was used; *Escherichia coli *XL-Blue cells (Evrogen, Russia) were utilized in plasmid cloning and production; *E. coli ΔlptD *(kindly provided by I.A. Osterman) was used as the target bacteria.

**Cloning the AMP genes**

The expression vector pGAP4 was constructed by replacing the PAOX1 promoter in the pPIC9k vector (Invitrogen) with the promoter PGAP sequence from vector pGAPZa (Thermo Fisher Scientific Inc., USA) using the HiFi DNA assembly kit (New England Biolabs, UK).

The nucleotide sequences of the AMP genes were optimized for production in *P. pastoris *cells using the GeneArt GeneOptimizer software (Thermo Fisher Scientific Inc.). The genes were synthesized by overlapping PCR and cloned into the expression vector pGAP4 using a HiFi DNA assembly kit (New England Biolabs). The cloned AMP genes and the gene encoding the yeast alpha-mating factor, which ensures the secretion of peptide molecules in the growth medium, lay within the same reading frame. During transport, the alpha-mating factor sequence was processed by KEX2 endopeptidase and the active peptide was released into the growth medium. The AMP library in the vector pGAP_AMP was linearized at the AvrII restriction site to be further transformed into yeast cells.

**Transformation of yeast cells**

The *P. pastoris *GS115 cells were transformed with the linearized plasmid library pGAP_AMP in accordance with the protocol reported in [19]. The transformed yeast cells were harvested from an RDB agar medium (1 M sorbitol, 20 g/L glucose, 13.4 g/L YNB, 0.4 mg/L biotin, 0.005 g/L of essential amino acids (L-glutamic acid, L-methionine, L-lysine, L-leucine, and L-isoleucine)) and cultured in an incubator at 30°C for 72 h.

**Evaluation of antimicrobial activity**

The transformed yeast library clones were seeded into cell culture dishes with a YPD agar medium (2% peptone, 1% yeast extract, 2% glucose, 100 mM potassium phosphate buffer pH 6.0, and 1.8% agar) and incubated at 30°C for 48 h. The target bacteria,* E. coli ΔlptD *GFP, were cultured overnight. Soft agar (0.8% tryptone, 0.5% yeast extract, 0.25% NaCl, and 0.5% agarose) was inoculated with the overnight culture of the target bacteria to a final concentration of 105 CFU/mL; the yeast colonies were overlaid with it and incubated at 37°C for 18 h until bacterial growth inhibition zones were formed.

**Analysis of the AMP production level in the liquid culture**

AMP-producing yeast strains were cultured in the YPD growth medium (2% peptone, 1% yeast extract, 2% glucose, and 100 mM potassium phosphate buffer pH 6.0) in shake-flasks at 30°C and 180 rpm overnight. Aliquots of the growth medium were sampled after 24, 48, and 72 h and analyzed by Tricine-SDSpolyacrylamide gel electrophoresis (PAGE), in accordance with the protocol described in ref. [20]. The AMP production level was assessed according to the intensity of protein bands after Coomassie staining.

**Peptide identification in clones exhibiting an antimicrobial activity**

Active yeast clones were grown on the selective RDB medium. The genomic DNA was extracted using lithium acetate and SDS according to the protocol reported in ref. [21]. The AMP genes were amplified by PCR using flanking primers: Forw 5’-TGCTAAAGAAGAAGGGGTATCTCTGGAGAAAAG- 3’ and Rev 5’-GAACTGAGGAACAGTCATGTCTAAGGCTACAAA- 3’. The PCR products were sequenced using the Sanger sequencing method; the peptide gene was identified by aligning the resulting nucleotide sequence to the sequence of the AMP genes in the panel.

**Extraction of yeast genomic DNA and sample preparation for next-generation sequencing**

The genomic DNA was extracted from the merged pool of transformed yeast clones according to the protocol described in ref. [21]. The AMP genes were amplified by emulsion PCR (ePCR) according to the protocol in [22] using the aforelisted primers. The resulting pool of PCR products was subjected to additional purification using VAHTS DNA Clean Beads (Vazyme, China).

**Next-generation sequencing**

The prepared PCR products were amplified using the REPLI-g Single Cell Kit (Qiagen, Germany). Sequencing was conducted using a HiSeq 2500 system, HiSeq PE Cluster Kit v4 cBot, and HiSeq SBSKit v4 (250 cycles) (Illumina, USA) in accordance with the manufacturer’s instructions.

## RESULTS

**Choosing the panel of antimicrobial peptides from the AMP databases**

Based on AMP databases such as APD3 [23] and DBAASP [24], we constructed a panel of AMPs exhibiting a prominent antimicrobial activity (*Table 1*).

**Table 1:** Structures of

Antimicrobial peptide	Amino acid sequence^*^	Length, aa	Structure type
TP4	FIHHIIGGLFSAGKAIHRLIRRRRR	25	β-sheet
Protegrin-1	RGGRLCYCRRRFCVCVGR	18	β-sheet
Magainin 1	GIGKFLHSAGKFGKAFVGEIMKS	23	α-helix
Melittin	GIGAVLKVLTTGLPALISWIKRKRQQ	26	α-helix
Mastoparan	INLKAIAALAKKLF	14	α-helix
Thanatin	GSKKPVPIIYCNRRTGKCQRM	21	β-sheet
HNP-1	ACYCRIPACIAGERRYGTCIYQGRLWAFCC	30	β-sheet
Tachyplesin-1	KWCFRVCYRGICYRRCR	17	β-sheet
Indolicidin	ILPWKWPWWPWRR	13	α-helix
Arminin 1a	KPWRFRRAIRRVRWRKVAPYIPFVVKTVGKK	31	α-helix

^*^The amino acid sequence, length and structure data were acquired from the APD3 [23] and DBAASP databases [24].

Validation using a panel of AMPs exhibiting different physicochemical characteristics is required to verify the ubiquity of the use of yeasts as a heterologous AMP producer. Based on this criterion, we chose AMP sequences with allowance for a relatively high antimicrobial activity, structural versatility, and length of the amino acid sequence. Hence, an AMP panel covering a broad range of structural templates was compiled.

**Creation of genetic constructs of the AMP panel and transformation of yeast cells**

**Fig. 2:**
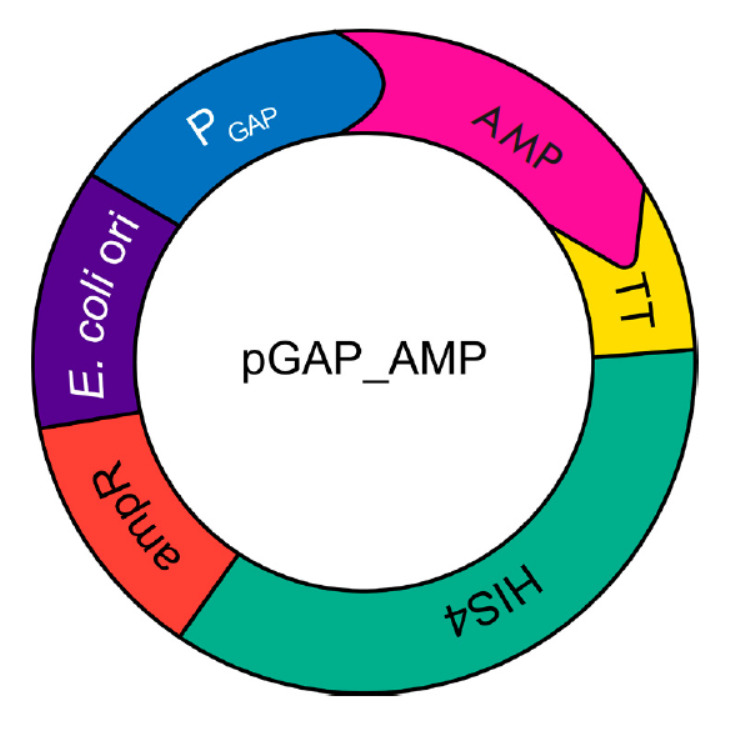
The scheme of the genetic construct for constitutive production of AMP

The nucleotide sequences of the genes encoding antimicrobial peptides were optimized using the GeneArt GeneOptimizer software (Thermo Fisher Scientific Inc.). The synthesized fragments were cloned into a yeast vector for secretory production of pGAP4_ AMP (*Fig. 2*). There was no need to add an inducer for target peptide production due to the presence of a strong constitutive promoter for the glyceraldehyde 3-phosphate dehydrogenase (GAP) gene. The resulting genetic constructs were transformed into yeast cells as a single pool. The yield of yeast clones produced using 1 μg of the plasmid library was 104; they were pooled together into a single library for further functional studies.

**Analysis of the representativity of the AMP genes from the panel**

**Fig. 3:**
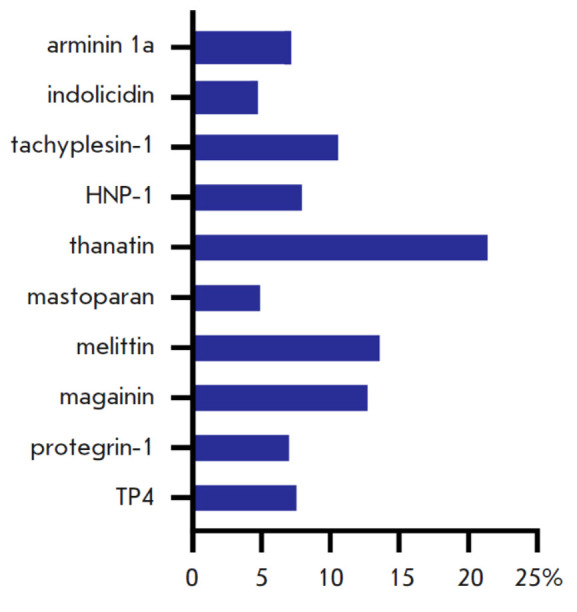
Analysis of AMP gene representation in a yeast library. The shares of the total number of sequences are displayed as a percentage on the X axis

The quantity of produced yeast clones was three orders of magnitude larger than the number of variants of the analyzed AMP genes, thus indicating that representativity of this library was sufficient. Extraction of total genomic DNA and next-generation sequencing were used to verify the presence of all the AMP genes from the panel in the library and establish their ratios. The sequencing data demonstrated that the library contained all the AMP genes from the panel (*Fig. 3*).

**Analysis of the antimicrobial activity of the library of AMP-producing yeasts**

**Fig. 4:**
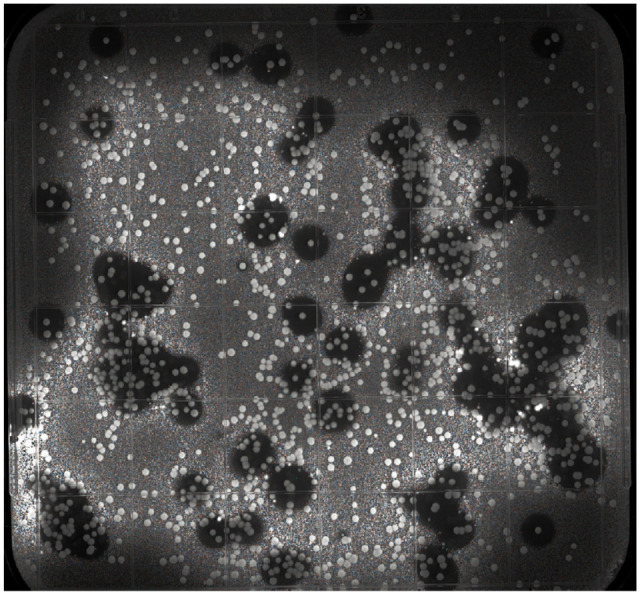
An assay of inhibition of the growth zones of the target bacteria Escherichia coli ΔlptD by yeast clones transfected with pGAP_AMP

Antimicrobial activity was tested by analyzing the formation of zones of growth inhibition of the target* E. coli ΔlptD* bacteria on the nutrient medium in Petri dishes (*Fig. 4*).

A total of ~ 3 000 clones were analyzed, covering the studied AMP library by more than two orders of magnitude. Thereby, the risk of overlooking a clone carrying the gene encoding any of the selected AMPs because of an insufficient number of analyzed clones was minimized. Genomic DNA was extracted in 55 clones. The region carrying the AMP gene was amplified and analyzed by Sanger sequencing. The analysis revealed that 37 clones carried the thanatin gene, while 18 clones carried the protegrin-1 gene.

Large zones of growth inhibition of the target bacteria were detected for active yeast clones, which could potentially overlap with the growth inhibition zones from other peptides. In order to rule out the potential loss of active clones, we additionally cloned a new pool of AMP genes, with the protegrin-1 and thanatin genes excluded. An analysis of clone activity in the shortened library revealed no new active candidates.

**Fig. 5:**
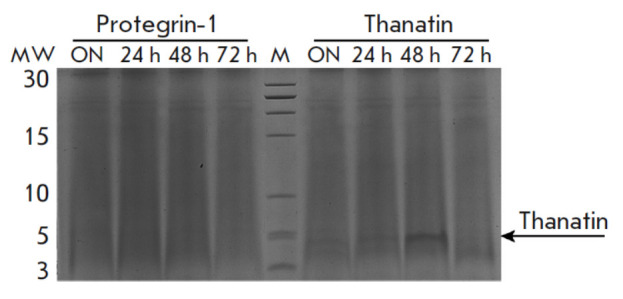
Tricine-SDS-PAGE electrophoregram. 24 h, 48 h, 72 h – culture media from protegrin-1 and thanatin producers taken at respective time points; ON – the overnight time point; M – protein molecular weight marker

The level of AMP production by active clones was also assessed according to band intensity in the SDS-PAGE analysis (*Fig. 5*).

The growth medium samples were characterized by a high level of recombinant thanatin production, as indicated by the presence of a clear band in the low-molecular-weight region of the electrophoregram. Meanwhile, the absence of respective bands in the culture media from protegrin-1 producers was demonstration that the production level of this peptide was low. However, despite the low level of protegrin-1 production, recombinant AMP-producing yeast clones exhibited a detectable level of antimicrobial activity, since this AMP *per se *has significant antimicrobial properties.

Our findings are indication that AMPs can be detected in this system both due to their high production level and according to their antimicrobial activity.

## DISCUSSION

Antimicrobial peptides (AMPs) are naturally abundant as defense and signaling molecules [25]. They mainly consist of 5–50 amino acid residues; positively charged and hydrophobic side chains are often predominant. A large group of AMPs has no specific protein target in bacterial cells, because they target the membrane or cause oxidative stress, thus suppressing the development of resistance to AMPs by bacteria [26]. Antimicrobial-resistant bacteria are also known to be substantially sensitive to AMPs [27]. Hence, AMPs are promising candidates for the role of alternative antimicrobial compounds for combating multidrug resistance.

The methylotrophic yeast *P. pastoris *is a convenient and cost-effective heterologous producer thanks to the availability of a vast pool of tools used in genetic engineering to produce a broad spectrum of protein molecules, as well as the low net cost of the required components of the growth medium. The cost of AMP synthesis and primary activity screening can be significantly reduced by detecting antimicrobial activity in a heterologous AMP producer. A library of yeast clones producing a panel of antimicrobial peptides was engineered in our study. Simultaneous activity assays of the yeast clones allowed us to compare the potential cytotoxicity of the selected peptides against eukaryotic cells and their specific activity. The protegrin- 1 and thanatin-producing clones were shown to exhibit a prominent antimicrobial activity.

Protegrin-1 is known to be characterized by high hemolytic activity and cytotoxicity [28], whereas thanatin, while specifically targeting bacteria, causes no marked hemolysis [29] and exhibits low cytotoxicity in mammals [30]. That can be one of the reasons behind the differences in the production levels of these AMPs observed in our study. The other peptide-producing strains from the panel exhibited no prominent antimicrobial effect against the target bacterium. The reason behind this can be the potential fungicidal activity of the respective AMPs against *P. pastoris* yeast cells. On the other hand, yeasts are eukaryotic organisms; therefore, AMPs targeting eukaryotic cell membranes via a nonspecific mechanism of action would be cytotoxic to yeast cells. In turn, this may reduce the production levels, thus leading to the lack of antimicrobial activity in the AMP-producing yeast. However, this effect can be used to exclude AMPs characterized by low selectivity against bacterial membranes and cytotoxic to eukaryotic cells. To be successfully detected by such screening, the potentially cytotoxic variants must be characterized by high specific activity.

Hence, it can be inferred that screening of active AMP variants by recombinant production in yeast cells allows one to select peptides with different characteristics. This system can be used to search for new templates for generating artificial diversity in AMPs and improving their pharmacokinetic properties.

## CONCLUSIONS

An AMP panel for generating a pool of recombinant antimicrobial producers was engineered in this study. The methylotrophic yeast *P. pastoris *was genetically modified to ensure secreted production of antimicrobial peptides. An analysis of the zones of growth inhibition of yeast clones demonstrated that producers of protegrin-1 and thanatin peptides exhibited the most prominent activity. An analysis of the production levels of protegrin-1 and thanatin revealed that thanatin content in the growth medium was higher than that of protegrin-1, thus indicating that there were different reasons for the manifested high activity of the yeast clones. For thanatin, this occurs due to its high production level, while being caused by high specific activity in the case of protegrin-1. Hence, by using yeast cells as AMP producers and generating a pool of cells exhibiting antimicrobial activity based on them, one can simultaneously analyze the antimicrobial properties of substances under the same conditions, thus reducing the time and cost of such research. This study demonstrated the potential of recombinant technologies in the development of strategies for massive screening of antimicrobial compounds.

## Abbreviations

AMP: antimicrobial peptide
GAP: glyceraldehyde 3-phosphate dehydrogenase
GFP: green fluorescent protein
NGS: next-generation sequencing
